# The Role of Inference in Diachronic Change in Conversational Implicature

**DOI:** 10.3390/bs16060869

**Published:** 2026-05-30

**Authors:** Jiayu Han, Yanfei Zhang

**Affiliations:** School of Foreign Languages and Literature, Shandong University, Jinan 250100, China; jiayuhan@mail.sdu.edu.cn

**Keywords:** inference, semantic change, conversational implicature, diachronic cognitive pragmatics

## Abstract

This study conducts a case study of *liǎnhóng* (脸红) to examine how inference works in the diachronic change in conversational implicature. It proposes three types of inference, namely ad hoc inference, entrenched inference, and conventionalized inference, with each contributing distinctively to semantic change. The findings reveal that inference is not principle-governed, but can be triggered by cognitive processes; conventions and context play distinct roles at different inferential stages; three types of inference derive different types of meanings within the inferential process, with ad hoc inference generating PCIs, entrenched inference converting PCIs into GCIs, and conventionalized inference ultimately developing GCIs into new coded meanings. This study elucidates the roles that different types of inference play in the diachronic change in conversational implicature, further demonstrating the intrinsic interconnection among three types of inference through corpus-based diachronic evidence.

## 1. Introduction

Since Grice (1989) proposes the classical theory of conversational implicature, inference has become a key topic in linguistic pragmatics. For Grice (1971, p. 58), “[S] meant something by x” is roughly equivalent to “[S] intended the utterance of x to produce some effect on the audience by means of the recognition of this intention.” In other words, Grice’s inferential model explains how the hearer infers the speaker’s conversational implicatures. In general, conversational implicatures are inferential in nature (Grice, 1989). Grice (1989) further divides conversational implicatures into generalized conversational implicatures (GCIs) and particularized conversational implicatures (PCIs), both of which are inferred in terms of four maxims of the Cooperative Principle (CP). GCIs are the implicatures closely associated with the use of specific forms of words in the absence of special context, assuming that the speaker follows conversational maxims. In contrast, PCIs are the context-dependent implicatures that arise when the speaker violates conversational maxims (Grice, 1989). Therefore, GCIs and PCIs are strictly divided.

Following Grice, Levinson (2000), Sperber and Wilson (1995), Carston (2002), and Bach (1994) propose different types of conversational inference for the computation of GCIs and PCIs. In linguistic pragmatics, conversational inference is studied both synchronically and diachronically. Synchronic studies, represented by Grice and his followers, hold that different types of conversational inference are strictly distinguished, resulting from the fact that there is a clear divide between GCIs and PCIs. But diachronic studies are very rare, especially from an empirical perspective. One of the key viewpoints of diachronic studies is that there is a diachronic change from PCIs into GCIs (Traugott & Dasher, 2002). Grounded in diachronic studies, this study is intended to investigate how conversational inference works in the diachronic change in conversational implicatures.

Throughout this study, “inference” refers exclusively to the processes of deriving meaning, while “implicature” and “coded meaning” refer to the products of these processes. Building on the diachronic pathway of semantic change (Traugott & Dasher, 2002; Schmid, 2015), this study proposes a diachronic cognitive pragmatic model, which involves three types of inference: ad hoc inference, entrenched inference, and conventionalized inference.

The remainder of this article is organized as follows. Section 2 reviews the post-Gricean accounts of inference. Section 3 presents the theoretical framework, and Section 4 outlines the research methodology. Section 5, Section 6 and Section 7 present a corpus-based case study, examining how each type of inference operates in the semantic change in *liǎnhóng*. Section 5 discusses the ad hoc inference of PCIs, Section 6 focuses on the entrenched inference of PCIs into GCIs, and Section 7 examines the conventionalized inference of GCIs into new coded meanings. Section 8 presents the discussion, followed by the conclusion in Section 9.

## 2. Inference in Post-Gricean Pragmatics

Inference plays a crucial role in the derivation of conversational implicatures (Levinson, 2000, 2024; Sperber & Wilson, 1995; Carston, 2002, 2019, 2021; Bach, 1984, 1994, 2017). Levinson (2000) identifies two types of inference: default inference and once-off inference. Default inference is “based on the choice of words or constructions or manner of production, which will, by tacit arrangement, signal specific messages” (Levinson, 2024, p. 20). Governed by the Q-, I-, and M-principles, default inference produces context-independent, conventionalized utterance-type meanings. The Q-, I-, and M-principles can be summarized as follows:

Q-principle

Speaker: Do not say less than is required (bearing the I-principle in mind);

Addressee: What is not said is not the case.

I-principle

Speaker: Do not say more than is required (bearing the Q-principle in mind);

Addressee: What is generally said is stereotypically and specifically exemplified.

M-principle

Speaker: Do not use a marked expression without reason;

Addressee: What is said in a marked way conveys a marked message.

(Levinson, 2000, pp. 76–137)

By contrast, once-off inference is “made in actual context by actual recipients with all of their rich particularities” (Levinson, 2000, p. 22), and involves a one-time analysis conducted by real recipients in specific context. Once-off inference relies on the violation of the maxims of Grice’s CP and derives utterance-token meanings, which are context-dependent and inherently non-conventional. This establishes a clear distinction between utterance-type and utterance-token meanings.

Relevance Theory rejects Levinson’s default inference, proposing that once-off inference, governed by the Principle of Relevance, can derive pragmatic meanings (Sperber & Wilson, 1995; Carston, 2002, 2009). Cutting across Grice’s GCI-PCI divide, Relevance Theory highlights the distinction between explicatures and implicatures, both of which are context-sensitive and derived by once-off inference. “An explicature is a combination of linguistically encoded and contextually inferred conceptual features” (Sperber & Wilson, 1995, p. 182), whereas “an implicature is the contextual assumption or implication which a speaker, intending her utterance to be manifestly relevant, manifestly intended to make manifest to the hearer” (Sperber & Wilson, 1995, pp. 194–195). Because Relevance Theory rejects the default inference, both explicatures and implicatures are context-dependent, non-conventional meanings derived through context-sensitive inference, without conventionalized interpretations.

Bach (1994, 1995) attempts to strike a balance between Levinson and Relevance Theorists, suggesting that the default inference is sensitive to context. He identifies two types of inference involved in deriving pragmatic meanings, namely default inference and once-off inference, both of which are contextually determined. Default inference simplifies the inferential step for both the speaker and the listener during communication, whereas once-off inference occurs in a specific context and does not simplify the inferential step (Bach, 1975). Another difference is that default inference is governed by the Take-for-Granted Principle (TFGP) and the Not-Worth-Considering Principle (NWCP), while once-off inference is governed by Grice’s CP. According to Bach (1984, p. 44), TFGP states that “its appearing to one that p justifies directly inferring that p provided that (a) it does not occur to one that the situation might be out of the ordinary, and (b) if the situation were out of the ordinary, it probably would occur to one that the situation might be out of the ordinary.” NWCP means that “if it occurs to one to do A, one is justified in directly deciding to do A, provided that (a) there occurs to one no thought of a reason to the contrary or of an alternative to A, and (b) such a thought probably would occur if it should” (Bach, 1984, p. 47). Furthermore, Bach (1994, 2017) suggests that default inference and once-off inference compute implicitures and implicatures, respectively. “Implicitures go beyond what is said, but unlike implicatures, which are additional propositions external to what is said, implicitures are built out of what is said.” (Bach, 1994, p. 137). Naturally, implicitures and implicatures are strictly distinguished.

Based on the above literature review, post-Griceans have developed distinct views on inference, as shown in Table 1.

In Table 1, three issues need to be further addressed. The first issue is about the relationship between inference and pragmatic principles. Post-Griceans (Levinson, 2000; Sperber & Wilson, 1995; Carston, 2002; Bach, 2017) propose that inference is governed by pragmatic principles. However, when Grice (2001) proposes the inferential model of conversational implicatures, he points out two types of reasoning in deriving conversational implicatures: “the hard way” and “the quick way.” The hard way of reasoning employs a “laborious, step-by-step procedure at least when we are in difficulties, when the course is not clear, when we have an awkward audience, and so forth” (Grice, 2001, p. 17), while the quick way of reasoning “is made possible by habituation and intention, is available to us, and the capacity for it (which is sometimes called intelligence, and is known to be variable in degree) is a desirable quality” (Grice, 2001, p. 17). In Grice’s view, the hard way of reasoning is driven by the CP, but the quick way of reasoning is governed by habituation or conventionalization. Thus, there is a clear need to investigate whether inference is principle-governed.

The second issue concerns the roles of conventions and context in the inference of conversational implicatures. Levinson (2000) argues that default inference is a process of conventionalization that arises independently of specific context, while Relevance Theorists (Sperber & Wilson, 1995; Carston, 2009) and Bach (2017) emphasize that inference is fundamentally context-dependent, rather than conventionalized. Therefore, whether inference is conventionalized^1^ or contextually determined is still hotly discussed in post-Gricean pragmatics.

The third issue is about the strict division between different types of inference. For instance, Levinson’s distinction between default inference and once-off inference shows that the former generates type meanings, whereas the latter derives token meanings; similarly, Bach’s distinction between default inference and once-off inference presents these two types of inferential processes as generating implicitures and implicatures, respectively. However, from a diachronic perspective, these inferential processes may be interconnected. As Grice (1989, p. 39) observes, “it may not be impossible for what starts life, so to speak, as a conversational implicature to become conventionalized”. In a study of the diachronic change in honorifics in Tamil, Levinson (1977) shows that the diachronic inferential process can cause PCIs to develop into conventional implicatures via GCIs.

In response to the three issues addressed above, the present study develops a diachronic cognitive pragmatic model of inference as its theoretical framework, and conducts a corpus-based analysis of the Chinese expression *liǎnhóng* (脸红) to investigate how different types of inference drive the diachronic change in conversational implicatures.

## 3. Theoretical Framework

This study draws on two usage-based frameworks: Traugott and Dasher’s Invited Inferencing Theory of Semantic Change (IITSC) and Schmid’s Entrenchment-and-Conventionalization Model (EC-Model). IITSC posits that semantic change begins when speakers invite hearers to derive meanings (Traugott, 2004, 2012). As Traugott and Dasher (2002, p. 5) explain, invited inference is “meant to elide the complexities of communication in which the speaker/writer evokes implicature and invites the addressee/reader to infer them,” and it operates at the outset of semantic change. Based on coded meanings, token meanings (PCIs) are individually inferred in different context, and a common inferential process is known as contextual extension, which constitutes a general mechanism involved in language change (Fischer & Traugott, 2016). Token meanings are gradually accepted by the speech community and conventionalized into type meanings (GCIs), which are finally semanticized into new coded meanings (Traugott, 2019).

Schmid (2015, 2020) proposes the entrenchment-and-conventionalization model, which integrates Traugott and Dasher’s framework from both individual and collective perspectives. The EC-Model highlights the usage-based and dynamic nature of linguistic systems, emphasizing the interaction between entrenchment and conventionalization (Verspoor & Schmid, 2024). According to Verspoor and Schmid (2024, p. 88), entrenchment is “the cognitive process that creates and sustains linguistic representations in the minds of speakers”, and conventionalization is the “social process that creates and sustains linguistic convention in a community of speakers”. Therefore, entrenchment is an individual activity, while conventionalization is a collective activity, and they can only interact through usage. Once entrenched, a meaning may become further conventionalized, accepted by all members of a speech community (Schmid, 2020).

**Figure 1:**
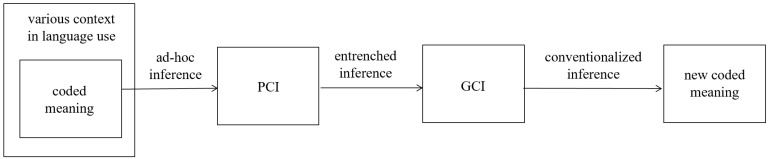
The model of diachronic cognitive pragmatics.

Drawing on both the Gricean inferential tradition and the usage-based theories discussed above, this study constructs a model of diachronic cognitive pragmatics (DCPM) to further explain different types of inference involved in the diachronic change in conversational implicatures (see Figure 1).

The DCPM identifies three types of inference: ad hoc inference, entrenched inference, and conventionalized inference. As illustrated in Figure 1, each type of inference plays a distinct role in the diachronic change in conversational implicatures, which proceeds from coded meanings through PCIs and GCIs to new coded meanings. Here, coded meanings refer to the stable semantic content shared across the speech community; PCIs are context-dependent token meanings derived through ad hoc inference on the basis of specific contextual information; GCIs are context-independent-type meanings routinized through repeated use and entrenched inference, yet not fully semanticized; new coded meanings are the stable semantic content that emerges when GCIs undergo conventionalized inference and come to be shared across the speech community. Ad hoc inference and conventionalized inference are proposed by Ehmer and Rosemeyer. According to them (Ehmer & Rosemeyer, 2018, p. 548), “the use of a construction in novel context leads to ad hoc inference by the hearer (corresponding to a PCI on the speaker side), repeated exposure to the same novel usage will lead to the conventionalization of this inference”. This study argues that a third type of inference, namely entrenched inference, operates in the diachronic change in conversational implicatures. Based on this, this study proposes a model illustrating how semantic change occurs through different types of inference, encompassing three key aspects.

Firstly, ad hoc inference derives PCIs. Ad hoc inference refers to the immediate process that hearers engage in to derive meanings when they encounter a construction in novel context (Ehmer & Rosemeyer, 2018). When speakers use a word or phrase in various contexts, they invite hearers to infer PCIs through ad hoc inference. This initial stage of the inferential process is characterized by the immediate and context-dependent nature of meaning interpretation, in which the hearers must actively infer new meanings based on specific contextual clues. Ad hoc inference is contextually triggered by metonymization, metaphorization and other cognitive processes. These processes serve as the fundamental drivers that allow speakers and hearers to extend meanings based on coded meanings. Metonymization refers to the formation of metonymy, which involves intra-domain mapping based on the relationship between one conceptual entity and another (Croft, 1993). Meanwhile, metaphorization means the formation of metaphor, which covers inter-domain mapping between the source domain and target domain in human cognition (Lakoff & Johnson, 1980). Additionally, it should be emphasized that the present study focuses on the two mechanisms as they emerge from the case of *liǎnhóng*. Other cognitive processes or semantic mechanisms, such as metaphtonymization and (inter)subjectification, may be relevant in other cases.

Secondly, entrenched inference is responsible for developing PCIs into GCIs. This intermediate inferential stage marks a crucial transition in which contextual meanings derived through ad hoc inference become inferentially entrenched through the repeated use of those meanings. At this stage, the frequency of use plays a vital role in entrenched inference. “With repeated use, a novel structure becomes progressively entrenched, to the point of becoming a unit; moreover, units are variably entrenched depending on the frequency of their occurrence” (Schmid, 2020, p. 3). The degree of entrenched inference correlates with use frequency, i.e., the more frequently a meaning or an associative pattern related to the meaning is used, the more deeply the meaning or pattern becomes inferentially entrenched in the individual mind. Entrenched inference gradually transforms what is initially an effortful and context-dependent interpretation of PCIs into an increasingly automatic and predictable GCIs. In sum, the more frequently a PCI is used, the more easily it is inferentially entrenched as a GCI in the individual’s mind.

Finally, conventionalized inference ultimately makes GCIs become new coded meanings. Conventionalized inference is similar to the conventional inference proposed by Morgan. According to Morgan (1977), conventional inference establishes the relation between linguistic form and literal meaning, which is arbitrary and based on the knowledge of language. He further distinguished two types of conventions: conventions of language (conventions of usage) and conventions about language (conventions of culture). In this study, conventions in conventionalized inference are the first type. Conventionalized inference refers to a stable process for interpreting meaning that develops when a novel construction is repeatedly used and accepted by the speech community (Ehmer & Rosemeyer, 2018). According to DCPM, conventionalized inference is a process of conventionalization, which is also driven by the frequency of repeated usage in language use. Unlike previous stages that operate primarily within the individual mind, conventionalized inference derives meanings that gain widespread acceptance across members of the speech community. In other words, through conventionalized inference, individually entrenched meanings (GCIs) achieve community-wide acceptance and become integrated into collectively new, conventionalized coded meanings. With the increased frequency and repetition, patterns of pragmatic inference become cognitive routines presumed to be shared by all members of a speech community. This shared routine represents a fundamental shift from individual interpretation to collective linguistic knowledge. Conventionalized inference occurs through extensive social interaction and communicative convergence, where members of a speech community gradually develop common interpretive expectations for specific meanings. Ultimately, conventionalized inference helps GCIs develop into new coded meanings.

In sum, the diachronic change in conversational implicature follows a path from ad hoc inference through entrenched inference to conventionalized inference, which just represents the missing link that the DCPM provides in explaining how PCIs (i.e., context-dependent pragmatic meanings) develop into new coded meanings over time, thereby integrating the model more seamlessly into the existing scholarship. The following sections will elaborate on how ad hoc inference, entrenched inference, and conventionalized inference each contribute to the diachronic change in conversational implicatures.

## 4. Data and Method

This study investigates the semantic change in *liǎnhóng* (脸红) as a case study to explore how different types of inference operate in the diachronic change in conversational implicatures. Following previous scholars who constructed specialized corpora drawn from large-scale corpora for various research purposes (Petré, 2016; Xu, 2019), this study builds a *liǎnhóng* corpus using data extracted from the CCL Corpus (http://ccl.pku.edu.cn:8080/ccl_corpus, accessed on 15 February 2026) (Zhan et al., 2019), a large-scale diachronic Chinese corpus.

The CCL Corpus comprises two sub-corpora: the Ancient Chinese Corpus, containing data from the Zhou Dynasty to the Qing Dynasty, and the Modern Chinese Corpus, covering data from the 1870s to the present. However, dividing the data into two periods is too general to capture the gradual semantic change in *liǎnhóng*, particularly given the extensive time span of the Modern Chinese corpus. Since the data in the CCL Corpus are arranged in chronological order, a finer periodization is feasible. Accordingly, this study divides the data into three periods: Ancient Chinese (Zhou Dynasty to Qing Dynasty), Modern Chinese (1870s to 1949), and Contemporary Chinese (1949 to the present).

A search for *liǎnhóng* in the CCL Corpus yields 6584 tokens spanning three periods. After excluding 1651 duplicates and invalid tokens, 4933 tokens remain for analysis. Invalid tokens refer to expressions in which the two characters *liǎn* (脸) and *hóng* (红) are syntactically and semantically separated and belong to different constituents (e.g., sù*liǎn hóng*méi, 素脸红眉, “unadorned face and red eyebrows”). Because the 4933 tokens constitute raw, unprocessed data, data-cleaning procedures are applied to remove elements irrelevant to the contextual analysis of *liǎnhóng*, including page number markers, special symbols, garbled characters, advertisements, emoticons, hyperlinks, and foreign-language texts.

To facilitate comprehension for non-Chinese-speaking readers, all examples are presented with interlinear glossing in accordance with the Leipzig Glossing Rules. Each example consists of four lines: the original Chinese sentence, Pinyin romanization, morpheme-by-morpheme glosses, and free translation. The grammatical abbreviations used in the morpheme-by-morpheme glosses throughout examples in this study are as follows: adverbial marker (ADV); ATTR (attributive marker); BA (*ba*-construction disposal marker); CL (classifier); EMPH (emphatic); GEN (genitive); INCH (inchoative); PFV (perfective aspect); PFX (prefix); PL (plural marker); POT (potential); PROG (progressive aspect); PRT (particle); RES (resultative complement marker); SFP (sentence-final particle); 1SG (first person singular); 2SG (second person singular). With these rules established, this study turns to the analysis of *liǎnhóng*. As illustrated in (1), *liǎnhóng* first appears in the Tang Dynasty:
(1)金缕浓薰百和香，
Jīnlǚnóngxūnbǎihéxiāng
gold-threadheavilyperfumehundred-blendincense
“(Her) gold-threaded garments (are) heavily perfumed with blended fragrances,”
*脸红*眉黛入时妆。

*liǎnhóng*méidàirùshízhuāng

face-redbrow-blackenter time-makeup

“(her) face turns red and darkened brows are done in fashionable makeup.”
(From *Quan Tangshi*《全唐诗》; the Ancient Chinese)

In (1), the woman’s face appears red due to the application of cosmetics. Here, *liǎnhóng* expresses its coded meaning, i.e., *liǎn fā hóng* (“the face turns red”). In the collected 4933^2^ tokens, 109 tokens convey the coded meaning, so the remaining 4824 tokens express various PCIs in different context. These 4824 tokens constitute the valid data and serve as the primary analytical focus of this study. The *liǎnhóng* corpus is constructed by compiling the contextual information for each valid token. Following Wilson and Kolaiti’s (2017) approach to analyze the contextual meanings (i.e., PCIs^3^) of “red eye”, the contextual scope is defined as six lines before and after the target word; when this scope proves insufficient for inferring the contextual meaning, it extends to ten lines.

Table 2 presents three sub-corpora of the *liǎnhóng* corpus. In this self-built corpus, the PCIs of *liǎnhóng* are inferred from specific context through the following analytic procedures. First, as stated earlier, two analysts delineated the context for each token, spanning six lines before and after its occurrence. Second, drawing on human cognitive processes and contextual information, the two analysts independently inferred the contextual meaning of each token in sequence. After completing the independent analysis, the analysts collaboratively reviewed and verified their interpretations. Any disagreements are resolved through discussion until a consensus is reached. Finally, to assess inter-rater reliability, Cohen’s Kappa is calculated based on the two analysts’ independent annotations of 4824 tokens. The result (κ = 0.833) indicates substantial agreement, demonstrating the reliability of the corpus analysis.

To systematically investigate how different types of inference operate in the semantic change in *liǎnhóng*, this study employs normalized frequency as its primary quantitative measure. Normalized frequency indicates the relative occurrence of linguistic phenomena in authentic language use, enabling comparative analysis across the corpora of varying sizes (Liang et al., 2010). It is adopted to quantify the diachronic distribution of conversational implicatures. The formula for normalized frequency is given by

$$normalized\;frequency=\frac{tokens\;of\;the\;target\;word}{total\;tokens\;of\;the\;corpus}\times 1,000,000$$

In this study, “tokens of the target word” represent the occurrences of specific PCIs. By calculating and comparing the normalized frequencies of different PCIs across ancient, modern and contemporary periods, this study traces the semantic change in *liǎnhóng* to examine how conversational implicatures change through different inferential stages. Section 5 introduces the ad hoc inference of PCIs.

## 5. Ad Hoc Inference of PCIs of *Liǎnhóng*

This section illustrates how ad hoc inference operates in the semantic change in *liǎnhóng*. As outlined in Section 3, ad hoc inference is triggered by two main cognitive processes: metonymization and metaphorization. How these cognitive processes trigger PCIs across different periods will be demonstrated in the following examples. In Ancient Chinese, 7 PCIs of *liǎnhóng* are derived through metonymization, as illustrated in (2)–(8). Specifically, (2), (3), and (4) reflect the metonymical transformation from the coded meaning “the face turns red” to various psychological states.
(2)红玉只装着和坠儿说话，也把眼去一溜贾芸：
HóngyùzhǐzhuāngzhehéZhuì’ershuōhuàyěbǎyǎnqùyīliūJiǎYún
HongyuonlypretendPROGwithZhui’ertalkalsoBAeyegoone-glanceJia Yun
“Hongyu pretended to be talking with Zhui’er, (while) also casting a glance at Jia Yun:”
四目恰相对时，红玉不觉*脸红*了。
sìmùqiàxiāngduìshíHóngyùbùjué*liǎnhóng*le
foureyejustmeetwhenHongyuunconsciouslyface-redPFV
“when (their) four eyes just met, Hongyu was unconsciously shameful.”
(From *Hong Loumeng*《红楼梦》; the Ancient Chinese)
(3)气得张月英*脸红*耳赤，赶过清风就是一刀。
qìdeZhāng Yuèyīng*liǎnhóng*ěr chìgǎnguòQīngfēngjiùshìyīdāo
anger RES Zhang Yueyingface-redear redrushtowardQingfengthenbeoneknife
“Zhang Yueying was so angered that (her) face and ears turned red, (she) rushed towards Qingfeng and (gave him) a slash of the knife.”
(From *Penggong An*《彭公案》; the Ancient Chinese)
(4)定哥叫贵哥进房中，要对他说些恁么话，
DìnggējiàoGuìgējìnfángzhōngyàoduìtāshuōxiēnènmehuà
DingGeaskGuiGeenterroominsidewanttohimsaysomesuchwords
“DingGe asked GuiGe to come into the room, (she) wanted to say something to him,”
却又*脸红*了不说，半吞半吐的束住了嘴。
quèyòu*liǎnhóng*lebùshuōbàn-tūn-bàn-tǔdeshùzhùlezuǐ
butagainface-redPFVnotsayhalf-swallow-half-spitADVrestrainPFVmouth
“but (she) blushed again and didn’t say (it), hemming and hawing, (she) holding her tongue.”
(From *Xingshi Hengyan*《醒世恒言》; the Ancient Chinese)

In (2), Hongyu pretended to converse with Zhui’er while using the conversation as a cover to steal glances at Jia Yun. When their eyes met, Hongyu unconsciously blushed, indicating shyness upon encountering Jia Yun’s gaze. In (3), the phrase “so angered that” explicitly marks anger as the cause, further corroborated by Zhang Yueying’s subsequent action of rushing towards Qingfeng and slashing him with a knife. In (4), DingGe intended to say something to GuiGe, but blushed and failed to speak, suggesting hesitation and unease. In these three cases, since “the face turns red”, through intra-domain mapping, constitutes a physiological response causally linked to internal psychological states, the physiological state stands metonymically for the psychological state. Thus, “shameful”, “anxious and angry”, and “nervous and uneasy” emerge as three PCIs of *liǎnhóng*.

In (5) and (6), *liǎnhóng* metonymically reflects physiological states caused by external factors, namely “drunk and intoxicated” and “injured or sick”.
(5)“我怎敢把你等闲厮觑？放心饮酒。”
wǒzěngǎnbǎnǐděngxiánsīqùfàngxīnyǐnjiǔ
1SGhowdareBA2SGcasuallyPFX look.atat-easedrink-wine
“How dare I look at you casually? Drink at ease.”
小童告过无礼，吃了几杯，
xiǎotónggàoguòwúlǐ,chīlejǐbēi,
young-servantapologizePFVrudenessdrinkPFVseveral cup
“The young servant apologized for being rude, drank several cups,”
早已*脸红*，道：“吃不得了。”
zǎoyǐ*liǎnhóng*dàochībùdéle
alreadyface-redsaydrinknotPOTPFV
“(his) face had already turned red, (and he) said: “(I) cannot drink anymore.”
(From *Erke Paian Jingqi*《二刻拍案惊奇》; the Ancient Chinese)
(6)金亨脸上肉伤青，打得*脸红*如血点；
Jīn Hēngliǎnshàngròushāngqīngdǎde*liǎnhóng*rúxuè diǎn
Jin Hengface-onfleshwound blue/bruisebeatRESface redlikeblood-spot
“Jin Heng’s face had flesh wounds and bruises, (he was) beaten till (his) face (turned) red like blood spots;”
虽然疼痛不作声。
suīránténgtòngbùzuòshēng
althoughpain/achenotmake-sound
“although (it was) painful, (he) did not make a sound.”
(From *Xiaobayi*《小八义》; the Ancient Chinese)

In (5), contextual clues such as “drink” and “drank several cups” establish a context of alcohol consumption. Excessive drinking induces a state of “drunk and intoxicated”, which is a general pathological state and manifests physiologically as facial redness. In (6), the contextual clues, including “flesh wounds and bruises”, “beaten”, “like blood spots”, and “painful”, collectively indicate that Jin Heng suffers physical harm, with his facial redness resulting directly from the beating. In both cases, “the face turns red” is a physiological state causally linked to general pathological states, enabling intra-domain mapping. Thus, “drunk and intoxicated” and “injured or sick” emerge as two PCIs of *liǎnhóng*.

By contrast, in (7) and (8), “the face turns red” metonymically extends to “cry until the face gets red” and “make efforts to exert force”, respectively.
(7)双翠合眉峰。泪华分*脸红*。
shuāngcuìhéméi fēnglèihuáfēn*liǎnhóng*
doublejade-greenjoineyebrow-peaktear-gleamdivideface-red
“The double jade-green (eyebrows) join at the eyebrow peaks. Tear gleams divide the rosy (cheeks).”
(From *Quan Songci*《全宋词》; the Ancient Chinese)
(8)这个上来摇一摇，涨得*脸红*；
zhègeshàngláiyáo yi yáozhàngde*liǎnhóng*
this-onecome-upshake-one-shakeswellRESface-red
“This one came up and shook (it), straining till (his) face turned red;”
那个上来拔一拔，挣得面赤。
nàgeshàngláibá yi bázhèngdemiàn chì
that-onecome-uppull-one-pullstruggleRESface red
“that one came up and pulled (it), struggling till (his) face turned red.”
(From *Shuoyue Quanzhuan*《说岳全传》; the Ancient Chinese)

Example (7) portrays facial redness caused by crying, while (8) illustrates facial flushing caused by physical exertion. Through the intra-domain mapping, *liǎnhóng* in these two examples conve*ys* “cry until the face gets red” and “make efforts to exert force” as PCIs.

The above seven examples show seven PCIs of *liǎnhóng* triggered by metonymization in Ancient Chinese, namely “shameful”, “anxious and angry”, “drunk and intoxicated”, “nervous and uneasy”, “injured or sick”, “cry until the face gets red”, and “make efforts to exert force”. These seven PCIs continue to be repeatedly used in Modern Chinese, with no new PCIs emerging during this period. However, PCIs of *liǎnhóng* are further enriched in Contemporary Chinese, where metonymization gives rise to seven additional PCIs: “awkward and embarrassed”, “excited and agitated”, “shocked and frightened”, “the face gets red due to temperature”, “happy and excited”, “the face gets red because of spicy food”, and “envious and jealous”. These seven PCIs are presented in (9)–(15).

Examples (9) and (10) demonstrate that *liǎnhóng* conveys two relatively mild psychological states: “awkward and embarrassed” and “envious and jealous”.
(9)小区经理们哄堂大笑，孙建冬差点*脸红*。
xiǎoqūjīnglǐmenhōngtáng-dàxiàoSūn Jiàndōngchàdiǎn*liǎnhóng*
districtmanagerPLroar-with-laughterSun Jiandongalmostface-red
“The district managers roared with laughter, Sun Jiandong almost blushed.”
(From *Dulala Shengzhi Ji*《杜拉拉升职记》; the Contemporary Chinese)
(10)本来自己背上不曾背着，
běnláizìjǐbèishangbùcéngbēizhe
originallyselfback-onnot-evercarryPROG
“Originally (it) had never carried (anything) on its back,”
看见别的蚂蚁背着，便*脸红*心热。
kànjiànbiédemǎyǐbēizhebiàn*liǎnhóng*xīnrè
seeotherantcarryPROGthenface-redheart-hot
“(but when it) saw other ants carrying (things), (it) then blushed and felt heart-hot.”
(From *Wangluo Yuliao*《网络语料》; the Contemporary Chinese)

In (9), the contextual clue “roared with laughter” indicates that Sun Jiandong became the object of collective laughter, creating a socially uncomfortable situation. His blushing response suggests a sense of awkwardness and embarrassment in the face of public attention. In (10), the ant “had never carried anything on its back”, yet upon “seeing other ants carrying things”, it blushes. This comparison implies envy. In both cases, the intra-domain mapping enables the physiological state to stand metonymically for the psychological state; thus, the PCIs of *liǎnhóng* in (9) and (10) are “awkward and embarrassed” and “envious and jealous,” respectively.

In addition to these relatively mild psychological states, *liǎnhóng* also conveys more intense psychological states, as illustrated in (11)–(13).
(11)测量目标对象在回应情绪激发问题时的
cèliángmùbiāoduìxiàngzàihuíyìngqíngxùjīfāwèntíshíde
measuretargetobjectatrespondemotiontriggerquestionwhenATTR
“Measure the target object’s (physiological activities) when responding to emotion-triggering questions”
呼吸、*脸红*反应、心跳频率、眼球运动
hūxī*liǎnhóng*fǎnyìngxīntiàopínlǜyǎnqiúyùndòng
breathingface-redreactionheartbeatfrequencyeyeballmovement
等生理活动。
děngshēnglǐhuódòng
etc.physiologicalactivity
“breathing, blushing reaction, heartbeat frequency, eye movement, etc.”
(From *Wangluo Yuliao*《网络语料》; the Contemporary Chinese)
(12)海云吓得*脸红*了。
Hǎiyúnxiàde*liǎnhóng*le
HaiyunscareRESface-redPFV
“Haiyun was so scared that (his) face turned red.”
他知道爸爸的脾气，不管谁作错了事，
tāzhīdàobàbadepíqibùguǎnshéizuòcuòleshì
heknowfatherATTRtemperno-matterwhodo-wrongPFVthing
“He knew (his) father”s temper, no matter who did something wrong,”
他总是不肯轻饶的。
tāzǒngshìbùkěnqīngráode
healwaysnotwillinglet-off-lightlySFP
“he would never let (them) off lightly.”
(From *Renmin Ribao*《人民日报》; the Contemporary Chinese)
(13)去丹霞山吧，让它给你一段
qùDānxiá Shānbaràngtāgěinǐyīduàn
goDanxia MountainSFPletitgiveyouoneCL
“Go to Danxia Mountain, let it give you a period of”
*脸红*心跳的时光。
*liǎnhóng*xīntiàodeshíguāng
face-redheart-beatATTRtime
“blushing and heart-pounding time.”
(From *Wangluo Yuliao*《网络语料》; the Contemporary Chinese)

In (11), “emotion-triggering questions” indicates a psychological state of emotional arousal. “So scared that” in (12) explicitly marks fear as the cause of facial reddening. In (13), “heart-pounding” evokes a psychological state of happiness and excitement upon encountering beautiful scenery, which manifests as a physiological state in which the face turns red. The physiological state “the face turns red” stands metonymically for internal psychological states through intra-domain mapping. Thus, *liǎnhóng* conveys “excited and agitated” in (11), “shocked and frightened” in (12), and “happy and excited” in (13).

Apart from the psychological states, *liǎnhóng* in Contemporary Chinese also denotes physiological states triggered by external factors, as presented in (14) and (15):
(14)冬天为什么会*脸红*？天一冷起来，
dōngtiānwèishénmehuì*liǎnhóng*tiānyīlěngqǐlái
winterwhywillface-redweatheroncecoldINCH
“Why does (one’s) face turn red in winter? Once the weather gets cold,”
我的脸就容易发烫，通红通红的。
wǒdeliǎnjiùróngyìfātàngtōnghóng~tōnghóngde
1SGGENfacetheneasilyemit-hotthoroughly-red. REDPRT
“my face easily becomes hot, turning completely red.”
(From *Wangluo Yuliao*《网络语料》; the Contemporary Chinese)
(15)小伙子对云南的重口味相当不适应，
xiǎohuǒziduìYúnnándezhòng-kǒuwèixiāngdāngbù-shìyìng
young-mantoYunnanATTRheavy-flavorquitenot-accustomed
“The young man was quite unaccustomed to Yunnan’s strong flavors,”
一点辣椒都会让他满头大汗、*脸红*脖子粗。
yīdiǎnlàjiāodōuhuìràngtāmǎntóu-dàhàn*liǎnhóng*bózicū
a-littlechilievenwillmakehimfull-head-big-sweatface-redneckthick
“even a little chili would make him sweat profusely, (with his) face red and neck thick.”
(From *Wangluo Yuliao*《网络语料》; the Contemporary Chinese)

In (14), “why does one’s face turn red in winter” and “once the weather gets cold” indicate that low temperature is the external factor for facial redness. “Even a little chili” in (15) identifies spicy food as the cause of the reddened facial appearance. In the above two cases, “the face turns red” is directly caused by external factors. The physiological state and its external cause belong to the same domain and are connected via the intra-domain mapping, and hence *liǎnhóng*, through metonymization, conveys “the face gets red due to the temperature” and “the face gets red because of spicy food”, respectively, in the above two examples.

By contrast, *liǎnhóng* generates only one PCI through metaphorization in Contemporary Chinese, “a lucky moment in online games”, as exemplified in (16).
(16)脸差有3星，脸好点就4星甚至5星了，
liǎnchàyǒusānxīngliǎnhǎodiǎnjiùsìxīngshènzhìwǔxīngle
facebadhavethreestarfacegooda-littlethenfourstarevenfivestarPFV
“(When your) luck is bad, (you) get 3 stars, (when your) luck is a bit better, (you) get 4 stars or even 5 stars,”
接个剔骨，怪就挂了，*脸红*的时候甚至剔骨都
不用。











jiēgètīgǔguàijiùguàle*liǎnhóng*deshíhoushènzhìtīgǔdōu
bùyòng











follow-upCLTigumonsterthenhangPFVface-redATTRtime even TiguEMPH
notneed











“(if you) follow up with a Tigu (skill), the monster dies, (when your) luck is really good, (you) don’t even need (to use) Tigu.”
(From *Wangluo Yuliao*《网络语料》; the Contemporary Chinese)

In (16), several gaming-specific terms establish the context: “stars” refers to the rating system for equipment quality, “face” is a gaming slang for luck, “Tigu” denotes a combat skill, and “monster” refers to enemies in the game. The sentence describes a situation in which a player with poor luck obtains only three-star equipment, whereas better luck yields four-star or five-star equipment. When the player achieves *liǎnhóng* (good luck), defeating a monster becomes so effortless that even “Tigu” is unnecessary. Based on this context, *liǎnhóng* acquires the PCI “a lucky moment in online games”. This PCI is derived through metaphorization via inter-domain mapping. At the physiological level, *liǎnhóng* denotes “the face turns red”; in the gaming context, red serves as a marker for high-quality and rare equipment. The mapping is grounded in the shared attribute of redness: just as the face turns red physiologically, rare equipment is visually marked by red in the game interface. Through this inter-domain mapping, *liǎnhóng* conveys “a lucky moment in online games” as one of its PCIs.

Based on the above analysis, the 4824 tokens produce 15 PCIs via metonymization and metaphorization in ad hoc inference. These 15 PCIs denote the contextual meanings identified in their initial occurrences of *liǎnhóng* within the corpus of 4824 tokens. Subsequently, some PCIs may develop into GCIs through entrenched inference driven by high-frequency repetition, further becoming new coded meanings via conventionalized inference. Alternatively, due to low repetition frequency or complete absence thereof, PCIs are still PCIs.

Among 15 PCIs, seven first appear in Ancient Chinese and then persist in Modern Chinese, while the rest of the eight emerge in Contemporary Chinese, as depicted in Table 3.

As shown in Table 3, metonymization triggers 14 PCIs, whereas metaphorization triggers only one PCI. According to the calculation results, normalized frequency for metonymization and metaphorization is 1532.048 pmc and 0.328 pmc, respectively. The two cognitive processes contribute differently to ad hoc inference. Compared with metaphorization, metonymization is a prominent cognitive process in ad hoc inference, as evidenced by its remarkably high normalized frequency. Although metaphorization generates only one PCI with a single token, and is thus almost negligible in terms of both low frequency of use and normalized frequency, the PCI derived through metaphorization remains indispensable to the semantic system of *liǎnhóng*. Although Sweetser (1990) proposes that metaphorization is the mechanism of semantic change, while Traugott (2018) argues that metonymization serves this role, this study contends that metonymization and metaphorization are not mutually exclusive in the semantic change.

## 6. Entrenched Inference of PCIs of *Liǎnhóng* into GCIs

In DCPM, normalized frequency serves as a metric for measuring how PCIs develop into GCIs through entrenched inference. The higher the normalized frequency of a PCI, the higher the degree of entrenched inference, and the more likely it is to convert into a GCI. Table 4 presents the normalized frequency of 15 PCIs mentioned in Section 5, with values arranged in descending order.

**Figure 2:**
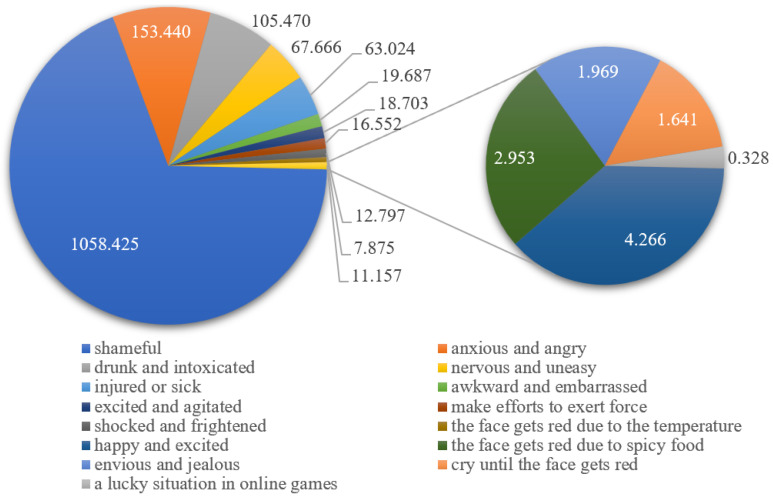
Proportion of the normalized frequency of 15 PCIs of *liǎnhóng*.

Drawing on the data in Table 4, Figure 2 provides a proportional representation of the normalized frequency of the 15 PCIs, allowing for a more intuitive comparison of their degree of entrenched inference in the *liǎnhóng* corpus.

In Figure 2, normalized frequencies of “shameful”, “anxious and angry”, “drunk and intoxicated”, “nervous and uneasy”, and “injured or sick” exhibit a substantial proportion of the distribution. These five PCIs with relatively high normalized frequencies are more likely to develop into GCIs in entrenched inference. Additionally, as depicted in Table 5, “shameful”, “anxious and angry”, “drunk and intoxicated”, “nervous and uneasy”, and “injured or sick” are repeatedly used across the three periods and undergo an “ancient → modern → contemporary” diachronic change. Consequently, these five PCIs show very high potential for developing into GCIs in entrenched inference.

Compared with the top five PCIs, the remaining 10 PCIs have lower normalized frequencies. “Awkward and embarrassed” has the highest normalized frequency among the ten, but its normalized frequency is only 19.687 pmc. By comparison, the last PCI, “a lucky situation in online games,” has a normalized frequency as low as 0.328 pmc. Additionally, the 10 PCIs—“awkward and embarrassed”, “excited and agitated”, “make efforts to exert force”, “shocked and frightened”, “the face gets red due to the temperature”, “happy and excited”, “the face gets red due to spicy food”, “envious and jealous”, “cry until the face gets red” and “a lucky situation in online games”—all first appear in Contemporary Chinese, with no repeated usage in the ancient and modern Chinese periods (see Table 5). Therefore, given their lack of repeated usage in the ancient and modern periods, and their lower or even extremely low normalized frequencies, the 10 PCIs are unlikely to develop into GCIs through entrenched inference. However, because “shameful”, “anxious and angry”, “drunk and intoxicated”, “nervous and uneasy”, and “injured or sick” are repeatedly used in the ancient, modern, and contemporary periods, and have relatively high normalized frequencies, the five PCIs become GCIs of *liǎnhóng* through entrenched inference.

## 7. Conventionalized Inference of GCIs into New Coded Meanings of *Liǎnhóng*

Five GCIs (i.e., “shameful”, “anxious and angry”, “drunk and intoxicated”, “nervous and uneasy”, and “injured or sick”) continue to undergo semantic change from the individual entrenched inference to the collective conventionalized inference. Conventionalized inference primarily operates on conventions of usage (Morgan, 1977), which depends on the repeated usage of GCIs. In other words, GCIs can be further conventionalized into new coded meanings through conventionalized inference. As mentioned in Section 3, the frequency of repeated usage affects the degree of conventionalized inference in GCIs, as measured by normalized frequency.

**Figure 3:**
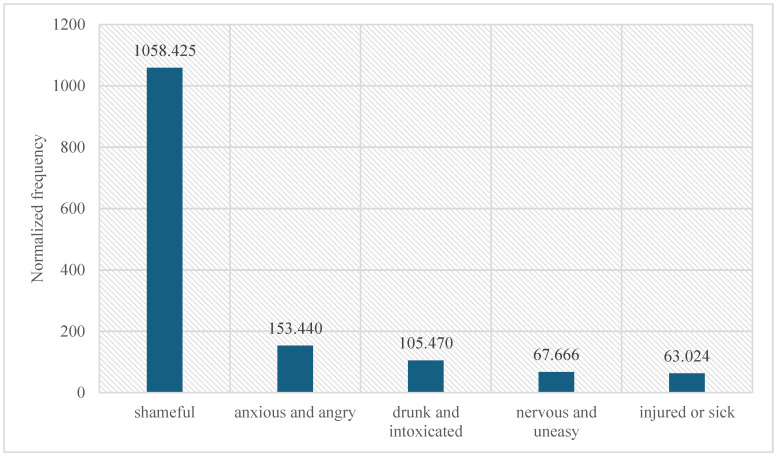
Comparison of the normalized frequency of GCIs.

Although “shameful”, “anxious and angry”, “drunk and intoxicated”, “nervous and uneasy”, and “injured or sick” become GCIs through entrenched inference, they differ in the degree of conventionalized inference. A higher normalized frequency indicates a higher degree of conventionalized inference and a stronger tendency to develop into new coded meanings. In contrast, a lower normalized frequency suggests a low degree of conventionalized inference and a weaker likelihood of such development. To enhance visual interpretation, Figure 3 compares the normalized frequencies of these five GCIs, making clear the degree of conventionalized inference. Their relative degrees of conventionalized inference, in descending order, are “shameful” > “anxious and angry” > “drunk and intoxicated” > “nervous and uneasy” > “injured or sick”. The high degree of conventionalized inference also indicates that the GCI is more likely to gain widespread acceptance across members of a speech community and become a new coded meaning.

What’s more, since the five GCIs undergo diachronic change across the ancient, modern, and contemporary periods, their different normalized frequencies in each period determine the corresponding degree of conventionalized inference (see Table 6).

**Figure 4:**
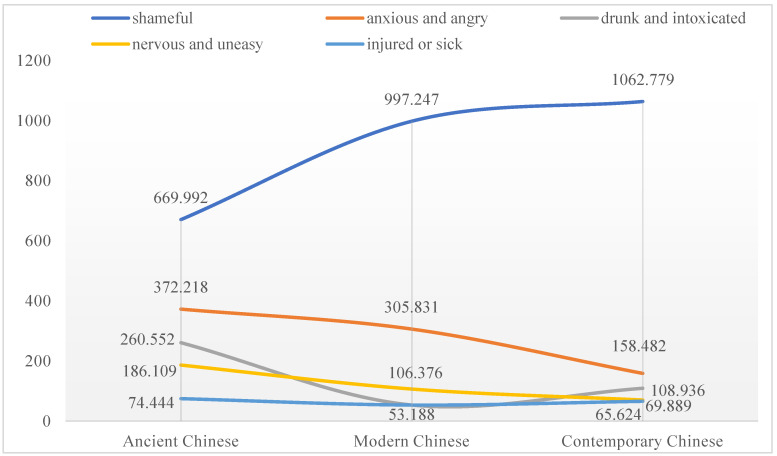
The trend of the normalized frequency of GCIs in the three periods.

Based on Table 6, the diachronic trend of GCIs across periods is further illustrated in Figure 4, which just reflects the degree of conventionalization of GCIs in the process of conventionalized inference. In Figure 4, “shameful” exhibits the highest normalized frequency and shows a consistent increase across all three periods (714.371 pmc < 997.247 pmc < 1062.779 pmc), indicating the highest degree of conventionalized inference and suggesting the greatest likelihood of developing into the new coded meaning of *liǎnhóng*.

The normalized frequency of “anxious and angry” ranks second only to “shameful” across the three periods, exceeding that of the remaining three GCIs, which indicates a degree of conventionalized inference lower than “shameful” but higher than the other three. However, the normalized frequency of “anxious and angry” does not exhibit continuous growth but instead shows a declining trend across the three periods (396.873 pmc > 305.831 pmc > 158.482 pmc). This pattern adversely affects its degree of conventionalized inference from the ancient to the contemporary period.

“Drunk and intoxicated” also shows a decline in normalized frequency across the three periods. Compared with the ancient period (277.811 pmc), its normalized frequency drops significantly in the modern period (53.188 pmc). Although there is a slight recovery in the contemporary period (108.936 pmc), the normalized frequency remains lower than that of the ancient period. This indicates that the degree of conventionalized inference for “drunk and intoxicated” decreases substantially from the ancient to the modern period. Despite some increase in the contemporary period, the overall degree of conventionalized inference remains weakened.

For the same reason, the normalized frequency of “nervous and uneasy” also exhibits a persistent decline trend across the three periods (198.436 pmc > 106.376 pmc > 69.889 pmc), representing a corresponding decrease in its degree of conventionalized inference across the three periods. The normalized frequency of “injured or sick” demonstrates a fluctuating decline. Its normalized frequency in the modern period (53.188 pmc) is lower than that in the ancient period (79.375 pmc). Although some increase is observed in the contemporary period (65.624 pmc), the normalized frequency remains below the ancient value, reflecting fluctuations in the degree of conventionalized inference across the three periods.

Although all GCIs undergo the complete inferential process across the ancient, modern, and contemporary periods, only the GCI “shameful” develops into the new coded meaning of *liǎnhóng*. On the one hand, the normalized frequency of “shameful” reaches 1058.425 pmc, whereas the highest normalized frequency among the remaining four GCIs, specifically “anxious and angry”, is merely 153.440 pmc. The former is nearly seven times the magnitude of the latter, indicating that “shameful” is far more prevalent than “anxious and angry”, “drunk and intoxicated”, “nervous and uneasy”, and “injured or sick” across different periods (as shown in Figure 4). On the other hand, although the “anxious and angry”, “drunk and intoxicated”, “nervous and uneasy”, and “injured or sick” are repeatedly used through the complete “ancient—modern—contemporary” semantic change process, their normalized frequencies exhibit an overall downward trend from a diachronic perspective. This suggests that their degree of repetition is generally diminishing. As mentioned above, “shameful” has the highest degree of conventionalized inference, resulting in the fact that it becomes a new coded meaning of *liǎnhóng*, and “anxious and angry”, “drunk and intoxicated”, “nervous and uneasy”, and “injured or sick” cannot become new coded meanings of *liǎnhóng*.

According to the Great Chinese Dictionary (vol. 6: 1386), two distinct meanings of *liǎnhóng* are documented, namely “the face turns red” and “shameful”, which just aligns with the findings of this study. This study suggests that the new coded meaning undergoes a complete inferential process, including ad hoc inference, entrenched inference, and conventionalized inference. Through this diachronic cognitive inferential process, the coded meaning of *liǎnhóng* gives rise to different PCIs under metonymization and metaphorization. PCIs systematically develop into new coded meanings by way of GCIs through repeated usage.

## 8. Discussion

By examining the diachronic semantic change in *liǎnhóng*, this study demonstrates that ad hoc inference, entrenched inference and conventionalized inference play distinct roles in driving the diachronic change in conversational implicatures. Ad hoc inference initiates the inferential process by deriving context-dependent PCIs through specific cognitive processes. Through high-frequency repetition, entrenched inference develops PCIs into GCIs, while conventionalized inference ultimately enables GCIs to become new coded meanings accepted by the entire speech community. In light of this inferential model in the corpus-based case study of *liǎnhóng*, the present research yields three major findings, which are discussed in detail below.

The first finding concerns the inference that it is not governed by principles. Post-Griceans generally assume that pragmatic inference is governed by principles—whether it is Q-, I-, and M-principles (Levinson, 2000, 2024), Principle of Relevance (Sperber & Wilson, 1995, 2025), or TFGP and NWCP (Bach, 1994, 2017). However, the DCPM model proposed in this study attempts to position metonymization and metaphorization as the cognitive triggers for ad hoc inference at the initial stage of the diachronic change in conversational implicatures. Similarly, entrenched and conventionalized inference do not necessarily involve principles; instead, they are triggered by the repeated usage of meanings. This finding demonstrates that the three types of inference (ad hoc inference, entrenched inference and conventionalized inference) are not governed by pragmatic principles, aligning with cognitive linguistic approaches that emphasize the role of conceptual mappings in meaning extension (Lakoff & Johnson, 1980; Traugott & Dasher, 2002). This invites a reconsideration of the universality of principle-based accounts and suggests that a usage-based, cognitively grounded framework may offer a more adequate explanation of how pragmatic meanings arise and undergo diachronic change. In this sense, the DCPM model contributes to the integration between cognitive linguistics and pragmatics.

The second finding is that conventions and context play complementary roles at different inferential stages. A key debate concerns whether implicatures are fundamentally context-dependent or conventionalized. Levinson (2000) argues that GCIs exhibit default, conventionalized interpretations independent of specific context, whereas Relevance Theorists (Sperber & Wilson, 1995; Carston, 2002) and Bach (1994) emphasize the context-sensitivity of all implicatures. The diachronic perspective adopted in this study suggests that, rather than being mutually exclusive, conventions and context operate at different stages of the inferential process. At the stage of ad hoc inference, hearers derive PCIs from specific contextual clues. As meanings become entrenched through repeated use, the role of context decreases while the role of conventions increases. At the stage of conventionalized inference, inference is primarily based on language conventions shared by the entire linguistic community, and context plays a significantly reduced role. Based on the corpus analysis of *liǎnhóng*, conventions and context fulfill their distinct roles in the diachronic change in conversational implicatures, demonstrating a clear division of labor across different stages of inferential processes. Such a dynamic theoretical perspective bridges the long-standing divide between contextualism and conventionalism, revealing that both represent different vantage points on the inferential process. This precisely confirms that context governs the first inferential stage, while conventions take over in the subsequent stages, demonstrating that context and conventions are complementary rather than opposed in the diachronic inferential process of conversational implicatures.

The third finding is that different types of inference convey distinct meanings and are closely connected. Post-Griceans typically draw strict boundaries between inference types and their products, for instance, Levinson’s (2000) distinction between utterance-type and utterance-token meanings, or Bach’s (1994) distinction between impliciture and implicature. These distinctions obscure the diachronic connections among different types of inference. The *liǎnhóng* data demonstrate that ad hoc inference, entrenched inference, and conventionalized inference consist of a developmental continuum. DCPM proposes that the three types of inference constitute a complete inferential process of conversational implicatures. Within this inferential process, ad hoc inference facilitates the development of coded meanings into PCIs; entrenched inference converts PCIs into GCIs; and conventionalized inference ultimately transforms GCIs into widely accepted new coded meanings. Therefore, the three types of inference operate at distinct stages during the diachronic change in conversational implicatures, with each deriving different types of meanings. It is worth noting, however, that different types of inference and their products (various types of meanings) constitute a dynamic developmental continuum rather than strictly distinct categories; different types of inference cannot be strictly distinguished (Elder & Haugh, 2024). Instead, they work collaboratively, forming a comprehensive inferential system, thereby highlighting the intricate connections among these types of inference.

By constructing the DCPM from a diachronic perspective, this study offers an analytical foundation for understanding the inferential processes underlying the diachronic change in conversational implicatures. Nevertheless, the present study leaves certain aspects open to further refinement. Focusing on the Chinese expression *liǎnhóng*, the present study aims to illuminate the distinct inferential stages through which such change proceeds, rather than establish cross-linguistic generalizations. Whether comparable expressions in other languages follow a similar inferential path, or exhibit varying degrees of entrenched and conventionalized inference, remains an open question requiring empirical verification. Preliminary cross-linguistic support for such inferential processes may be drawn from Spanish historical pragmatics, where studies of address forms, speech acts, and (im)politeness strategies similarly document a gradual progression from context-dependent to conventionalized pragmatic patterns (Cruz Volio, 2017; Gancedo Ruiz, 2021; Iglesias Recuero, 2022; Albitre Lamata, 2025). While those studies primarily examine the diachronic change in pragmatic patterns in Spanish, the present study focuses on tracing the inferential processes underlying the diachronic change in conversational implicatures. In other words, previous studies centers on patterns or constructions, but our study mainly deals with the semantic change in conversational implicatures.

Furthermore, a limitation of the present study lies in what frequency-based evidence can reveal. Although the DCPM accounts for the process by which PCIs develop into new coded meanings through entrenched inference and conventionalized inference, frequency data alone cannot determine whether the diachronic distribution of usage frequency reflects lexicalization, grammaticalization, or an interaction between these two processes. This is not merely a methodological caveat but a substantive constraint on the generalizability of the present findings. Accordingly, conclusions drawn from frequency data document in this study are regarded as preliminary and hypothesis-generating rather than definitive. Future research could address this by adopting a grammaticalization-informed approach that traces the parallel change in syntactic structure and semantic content in *liǎnhóng*, thereby enabling a more rigorous investigation of the extent to which frequency reflects deeper grammatical restructuring. Moreover, the absence of an analysis of external lexical competition means that other constructional or sociolinguistic influences cannot be fully assessed. Future research should also consider external lexical competition as a key variable, referring to the degree to which the semantic space for a given meaning becomes occupied by more specialized vocabulary, as this factor may substantially interact with the internal cognitive variables already incorporated in the present model.

## 9. Conclusions

This study examines the semantic change in *liǎnhóng* as a case study to investigate how different types of inference function in the diachronic change in conversational implicature. Ad hoc inference transforms the coded meaning into PCIs, entrenched inference develops PCIs into GCIs, and conventionalized inference ultimately converts GCIs into new coded meanings. The clear distinction between different types of meanings limits the comprehensive understanding of the relationship between GCIs and PCIs. Therefore, this study constructs DCPM from a diachronic perspective and positions it as a specialized diachronic extension. Moreover, ad hoc inference and entrenched inference are context-driven, whereas conventionalized inference is governed by the conventions of language use; in this regard, context and conventions each play their respective roles at different stages of inferential processes. It is clearly indicated that three different types of inference diachronically make PCIs entrenched into GCIs, which are also conventionalized into new coded meanings. This study sheds light on the role of inference in the diachronic change in conversational implicatures.

## Figures and Tables

**Table 1:** Different views on inference in post-Gricean pragmatics.

	Levinson	Relevance Theorists	Bach
Types of inference	Default/Once-off inference	Once-off inference	Default/Once-off inference
Governing principle	Q-, I-, M-Principles; Grice’s CP	Principle of Relevance	TFGP&NWCP; Grice’s CP
Meaning distinction	Type meanings/Token meanings	Explicature/Implicature	Impliciture/Implicature
Role of context	No	Yes	Yes
Role of conventions	Yes	No	No

**Table 2:** Three sub-corpora of the *liǎnhóng* corpus.

Sub-Corpus	Frequency of *Liǎnhóng*	Tokens of Sub-Corpus
Ancient Chinese Corpus	44	25,197
Modern Chinese Corpus	117	75,205
Contemporary Chinese Corpus	4663	3,047,671

Adopting the CCL Corpus’s binary historical division would be too general to capture the gradual semantic change in *liǎnhóng*, especially given the extensive time span of the Modern Chinese sub-corpus. Since the CCL data are arranged chronologically, a finer periodization is feasible. Accordingly, this study divides the data into three periods: Ancient Chinese (Zhou Dynasty to Qing Dynasty), Modern Chinese (1870s to 1949), and Contemporary Chinese (1949 to the present). According to the three periods, the *liǎnhóng* corpus is organized into three corresponding sub-corpora.

**Table 3:** PCIs derived by two cognitive processes diachronically in ad hoc inference.

Historical Period	PCI	Cognitive Progress
Ancient Chinese	shameful	Metonymization
	anxious and angry	
	drunk and intoxicated	
	nervous and uneasy	
	injured or sick	
	cry until the face gets red	
	make efforts to exert force	
Modern Chinese	shameful	
	anxious and angry	
	drunk and intoxicated	
	nervous and uneasy	
	injured or sick	
	cry until the face gets red	
	make efforts to exert force	
Contemporary Chinese	awkward and embarrassed	
	excited and agitated	
	shocked and frightened	
	the face gets red due to the temperature	
	happy and excited	
	the face gets red due to spicy food	
	envious and jealous	
	a lucky moment in online games	Metaphorization

**Table 4:** Normalized frequency of PCIs in the *liǎnhóng* corpus.

PCI	Frequency	Normalized Frequency
shameful	3332	1058.425
anxious and angry	483	153.440
drunk and intoxicated	332	105.470
nervous and uneasy	213	67.666
injured or sick	198	63.024
awkward and embarrassed	60	19.687
excited and agitated	57	18.703
make efforts to exert force	52	16.552
shocked and frightened	39	12.797
the face gets red due to the temperature	24	7.875
happy and excited	13	4.266
the face gets red due to spicy food	9	2.953
envious and jealous	6	1.969
cry until the face gets red	5	1.641
a lucky situation in online games	1	0.328

**Table 5:** The historical distribution of PCIs of *liǎnhóng*.

PCI	Historical Period
shameful	Ancient/Modern/Contemporary
anxious and angry	Ancient/Modern/Contemporary
drunk and intoxicated	Ancient/Modern/Contemporary
nervous and uneasy	Ancient/Modern/Contemporary
injured or sick	Ancient/Modern/Contemporary
awkward and embarrassed	Contemporary
excited and agitated	Contemporary
make efforts to exert force	Contemporary
shocked and frightened	Contemporary
the face gets red due to the temperature	Contemporary
happy and excited	Contemporary
the face gets red due to spicy food	Contemporary
envious and jealous	Contemporary
cry until the face gets red	Contemporary
a lucky situation in online games	Contemporary

**Table 6:** Distribution of GCIs across the three periods in the *liǎnhóng* corpus.

	Ancient Chinese		Modern Chinese		Contemporary Chinese	
GCI	Frequency	Normalized Frequency	Frequency	Normalized Frequency	Frequency	Normalized Frequency
shameful	18	714.371	75	997.247	3239	1062.779
anxious and angry	10	396.873	23	305.831	483	158.482
drunk and intoxicated	7	277.811	4	53.188	332	108.936
nervous and uneasy	5	198.436	8	106.376	213	69.889
injured or sick	2	79.375	4	53.188	200	65.624

## Data Availability

The data presented in this study are available on request from the corresponding author.

## Notes

1 In Spanish historical pragmatic studies of address forms, speech acts, and (im)politeness strategies, context-dependent pragmatic patterns are proven to be conventionalized through inferential processes (Cruz Volio, 2017; Gancedo Ruiz, 2021; Iglesias Recuero, 2022; Albitre Lamata, 2025). For instance, the process of diachronic change from *vuestra merced* (lit. ‘your grace’) to *usted* (lit. ‘you’) (Iglesias Recuero, 2022) serves as an instructive illustration, indicating that no strict boundary exists between different types of inference.

2 Of the 4933 tokens collected, 109 express the coded meaning of *liǎnhóng*, while the remaining 4824 tokens convey PCIs. As this study aims to examine how PCIs develop into new coded meanings through different types of inference, it selects these 4824 tokens as the object of analysis.

3 In this study, PCIs are firstly identified by metonymization, metaphorization and other cognitive processes in different context, and then the frequency of repeated usage of PCIs is calculated. Some PCIs with high frequency of repeated usage can become GCIs, which is different from the views mentioned in the introduction part.
